# Aware but confused: conflicted between individual and collective responsibility. A grounded theory study of norms and organisational structures relating to sexual harassment among university students in southern Sweden

**DOI:** 10.1080/17482631.2025.2471667

**Published:** 2025-02-26

**Authors:** Jack Palmieri, Maria Emmelin, Pia Svensson, Anette Agardh

**Affiliations:** Social Medicine and Global Health, Department of Clinical Sciences, Lund University, Malmö, Sweden

**Keywords:** Sexual harassment, university students, power, social cognition theory, grounded theory

## Abstract

**Purpose:**

Sexual harassment in contexts of higher education is a well-documented problem with far reaching consequences for individuals and organizations. Questions remain about how sexual harassment is conceptualized and what implications these conceptualizations have for designing programmes to prevent and respond to sexual harassment in university settings. This study aimed to understand how students conceptualize sexual harassment, focussing on the influence of perceived norms and organizational structures as explanatory mechanisms.

**Methods:**

This grounded theory study utilized seven focus group discussions to collect data from students at Lund University, Sweden.

**Results:**

The analysis yielded one core category, “Aware but confused: conflicted between individual and collective responsibility”, supported by four categories reflecting different elements of conceptualizing and responding to sexual harassment. Sub-categories captured properties and dimensions of these categories along the continuum of individual to collective responsibility. The findings reflected an awareness among students of the occurrence of sexual harassment but confusion over definitions and assignment of responsibility. This confusion could have serious consequences for willingness to report cases of sexual harassment.

**Conclusion:**

Building trust in the university system requires establishing common understandings of sexual harassment, clear and accountable pathways for reporting, and transparency of outcome when reports are made.

## Introduction

Sexual harassment (SH) in contexts of higher education is a persistent and damaging problem. A recent systematic review of the literature highlights a global prevalence of 25% among female students (Bondestam & Lundqvist, 2020), with a large-scale study in Norway presenting prevalence figures of 21.6% for the same population (Sivertsen et al., 2019) and a study at Lund University in Sweden reporting 26.8% of female and 11.3% of male students exposed to sexual harassment during their time at the university (Agardh et al., 2022). Exposure to sexual harassment has been linked to a large number of severe outcomes for individuals both in terms of their physical and mental health and wellbeing (McDonald, 2012) and in terms of motivation and study progression (Wolf et al., 1991).

The existing global research on sexual harassment in higher education is dominated by quantitative cross-sectional studies (Bondestam & Lundqvist, 2020) that tend to focus on developing empirically founded definitions of sexual harassment that lack theoretical perspectives (Fitzgerald, 1990). Indeed, a recent scoping review of women students’ experiences of sexual violence globally (a broad categorization that included experiences of sexual harassment) highlighted the dearth of qualitative research on lived experiences of sexual harassment in higher education as well as the importance of understanding the organizational environment and its role in reducing stigma, promoting awareness and encouraging help-seeking behaviours (Tarzia et al., 2023).

Among the qualitative literature that does exist, most of the studies have been conducted outside of Scandinavia. Large differences in the university settings between these geographical areas complicate transferability of the results.

In the global research, one study conducted among graduate students in the USA explored the culture of silence around reporting sexual harassment (Bloom et al., 2021). The study’s results showed the importance of recognizing power relationships between perpetrator and victims, a lack of trust in university structures and their accountability and uncertainty regarding the incidence of sexual harassment in their study and work environment.

Another mixed-methods study conducted in the UK explored the conceptualization of sexual harassment as a “student problem” and how this could hide the role that the university culture context could play. The study’s results highlighted the importance of a conducive context marked by gender inequality as the scaffolding for sexual harassment to occur. The study also indicated the need to adopt an institutional and structural level focus in analysing and responding to sexual harassment in university settings (McCarry & Jones, 2022).

Similarly, in the Swedish context, very few peer-reviewed studies exist. Among those included in a recent review (Vetenskapsrådet, 2018), one study explored the effect of discourse on the construction of sexual harassment in a Swedish university. The results showed the importance of recognizing variability and experience as critical tools for challenging normative views of sexual harassment in a university setting (Bondestam, 2004b).

A number of PhD theses have been published on the topic of sexual harassment in Swedish higher education. These explore gender-offensive behaviour among students and staff in academic environments (Andersson, 2007), sexual harassment and gender among PhD students and the challenges of the Swedish gender discourse (Carstensen, 2004) and the broader aspects of gender equality in academia including policy programmes for gender equality and sexual harassment (Bondestam, 2004a).

Although no consensus exists over any single cause of sexual harassment, there are many different theoretical perspectives and models that seek to define and explain sexual harassment (Pina et al., 2009). These theories can broadly be divided into single or multifactor theories that recognize that sexual harassment does not occur in isolation and is most accurately attributable to a number of different factors (Pina et al., 2009).

Preventing sexual harassment requires an understanding of individual factors and organizational structures related to how sexual harassment is conceptualized. Despite this, much of the research focuses on tertiary prevention in the form of systems for supporting redress for individuals who have been exposed to sexual harassment (Fedina et al., 2018). In addition, many existing studies frame sexual harassment among the student population as a “student problem” (McCarry & Jones, 2022). The individualization of the question hides the structural, institutional and relational aspects of organizations that could be pivotal for understanding and preventing sexual harassment (Ollo-López & Nuñez, 2018). This includes the norms surrounding sexual harassment that might affect interventions such as bystander approaches to prevention (Mujal et al., 2021).

The four level of analysis model in the area of social cognition (Doise, 1986) is a theoretical framework that has been developed to allow a holistic examination of complex phenomena. This model posits that social phenomena can best be understood by examining them at different levels and exploring the interconnection between the levels (termed articulation) (Doise, 2012). The four level model encompasses a focus on the intraindividual level that examines the way personal experiences, beliefs and attitudes shape one’s own understanding; the interindividual level that reflects how relationships and communication through social networks can shape perception and behaviour; the positional level that deals with group dynamics in the context of social categories and positions in a given framework; and the ideological level that examines how ideologies, social representations of norms and institutional frameworks can influence understanding and actions (Doise, 1986). Utilising the logic of the four level of analysis model for conceptual and empirical analysis as sensitizing concepts supported the current researchers in identifying directions in which to look without limiting or steering the data collection or analysis (Blumer, 1954).

The existing research into sexual harassment among students in higher education highlights the need to lift the focus from a narrow, single-level perspective of it being a “student problem” (McCarry & Jones, 2022) and to explore the phenomenon in such a way that encapsulates conceptualization and context (Hagerlid et al., 2023). Thus, the four level of analysis model can provide a useful theoretical perspective.

The current qualitative study seeks to understand what the concept of sexual harassment means for students in a university setting, focussing on the influence of perceived norms and organizational structures.

## Method

### Study design

To explore university students’ understandings of sexual harassment, a qualitative study design with grounded theory methodology was used. Focus group discussions (FGDs) were considered appropriate for data collection as they provide researchers with access to group dynamics and conversations that can articulate norms and attitudes towards specific topics such as organizations (Kitzinger, 1995). FGDs have also been shown to be useful for eliciting context-specific language when discussing sensitive issues, for reflecting social conventions as well as for possibilities for depersonalizing the discussion (Jordan et al., 2007).

A grounded theory approach was employed due to the fact that it is a systematic research methodology useful for constructing an explanatory model or theory about a phenomenon of interest (Corbin & Strauss, 2015). Since the aim was to generate an explanation of behaviours and perspectives when viewed through an organizational lens and to generalize (theoretically) to a broader context (Singh & Estefan, 2018), grounded theory as described by Corbin and Strauss (Corbin & Strauss, 2015) was the specific approach selected.

### Study setting

The study was conducted among students at Lund University in southern Sweden. Lund University is a university with around 30,000 students and 8,000 staff across 9 faculties and 4 campuses. The university is a global institution, with 28% of students and 37% of staff from over 130 countries outside of Sweden. Lund University has a thriving student social life, with many activities run by student associations or “nations”. These 13 “nations” are large social clubs mostly run by students that organize social activities. Some nations also have accommodations where students can live (Lund University, 2023).

Data collection was part of “Tellus”, a university-wide project aimed at strengthening prevention and response to sexual harassment for students and staff. Initiated in 2018, Tellus was a response to the #metoo movement and the resulting discussions within academia. The overall purpose was to examine exposure to sexual harassment at an academic workplace/study environment and its health-related consequences for the individual and to identify potentially relevant contributing and protective factors at the individual and contextual level. The project encompassed qualitative and quantitative studies. Key results of the survey data outlining the magnitude and characteristics of sexual harassment in this setting can be found in an overview article (Agardh et al., 2022). Previous research has also been conducted on the role that the psychosocial study environment (and specifically high strain situations) plays in sexual harassment in this setting (Palmieri et al., 2023).

### Sampling and data collection

Sampling of participants for this study was strategic, aiming to reach a diversity of students interested in discussing issues related to sexual harassment at Lund University. To ensure a broad range of attitudes and opinions, the invitation was kept purposefully broad and directed to all students at Lund University with no requirement of personal experience of sexual harassment. Invitation for participation in the focus group discussions was advertised university wide utilizing physical posters distributed to all campuses, information points, and student newspapers and media. Students interested in participating in the study contacted the project via email and were then offered times and locations for participation in a group discussion. In total, 28 individuals participated across 7 group discussions, with group size ranging from 3–6 participants per group. To capture the discourse around sexual harassment between different types of students, group composition was mixed, with male, female and non-binary students, and bachelor and master students in the same groups.

A semi-structured discussion guide was developed by the main authors in collaboration with reference groups representing students, student organizations, and the student unions as well as the university management and HR department. This guide included questions on students’ understandings of the occurrence of sexual harassment, what sexual harassment means and in what situations sexual harassment occurred. The guide also covered questions about the study environment and expectations for the future response from the university. The guide was pretested with staff from the neighbouring Malmö University. Due to the process of constant comparison, where data collection and analysis occur simultaneously/cyclically (Corbin & Strauss, 2008), the wording used to present the topics to the groups was revised and developed periodically throughout the data collection based on the emerging knowledge.

All group discussions were conducted in a quiet and private room at Lund University. Six of the group discussions were moderated by the first author of this article (JP), with the last author (AA) as co-moderator, and one FGD was moderated by the last author (AA) with the first author (JP) as co-moderator. Three group discussions were conducted in Swedish and four in English. Participants could choose freely whether to attend FGDs in Swedish or English, and the language chosen did not necessarily reflect the person’s language or country of origin. More details about the discussion groups’ constitution can be found in Table I.

**Table I.** Characteristics of focus group discussion participants.

FGD#	Total # participants	#females	#males	#neither female nor male	Language
1	4	3	1		Swedish
2	6	4	2		English
3	4	4			English
4	4	1	3		Swedish
5	3	2	1		English
6	3	2		1	Swedish
7	4	2	2		English

### Data analysis

All FGDs were digitally recorded. In addition, the first author (JP) maintained a reflexive journal where notes pertaining to each individual group discussion were recorded directly after the FGD had been completed. The reflexive journal contained thoughts on the immediate group discussion completed, as well as a development of core ideas that formed as a culmination of the FGDs to date. A seating diagram showing how the participants and moderators were seated in each discussion was also prepared in line with recommendations for FGD research (Krueger & Casey, 2015). These seating diagrams were used to facilitate the transcribing process and to support the analysis of patterns in responses and narrative threads within the broader discussions. The moderator and co-moderator (JP and AA) discussed each FGD, commenting both on the content of the discussions as well as the language used and type of probes employed. This allowed a constant development of the researchers’ language. Following the first three FGDs, a meeting was held between three of the authors (JP, AA and ME) to discuss the data collected in more detail and suggest developments of the collection process. This interrelatedness of data collection and analysis are core tenets of grounded theory (Corbin & Strauss, 2015).

**Figure 1.**
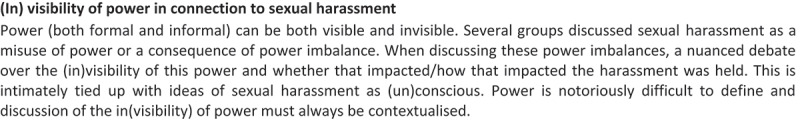
Example of a memo describing early reflections of power and its (in)visibility.

Each interview was transcribed verbatim by an experienced transcriber connected to the research team, and transcripts were cross-checked by the first author (JP). Each transcript was then read through from beginning to end alongside the reflexive journal comments for that discussion. Open coding was first used to “open up” the data (Corbin & Strauss, 2015). Through this process, concepts were identified on different levels. Memos were used to explore the first author’s (JP) thoughts about the coded material and to capture the “mental dialogue” between the author and data. These memos were discussed with the other authors in the study. An example of a memo can be seen in Figure 1. Properties and dimensions of the concepts were also captured in the memos to expand upon their definition and better describe the concepts. At the end of this first step, emerging concepts were used to focus the analysis and return to the original data set to explore these conceptual insights as a form of theoretical sampling (Conlon et al., 2020), aided by the expansion of memos. From these expanded codes, categories were formed, and one core category developed using constant comparison methods. The analysis continued until a “theoretical sufficiency” was achieved where a depth of understanding was reached that allowed the researchers to build a theory based on categories suggested by data (Dey, 1999). Finally, a theoretical model was constructed and agreed upon by all authors.

### Ethical considerations

The Swedish Ethical Review Authority granted approval to the study protocol (number: 2018/350). Prior to the FGDs, each participant was provided with information about the research study and given the opportunity to ask questions and receive clarifications. Participants were especially informed about the format of the FGDs in that researchers can only guarantee confidentiality on behalf of the research team but not in relation to group participants. Once participants’ questions had been answered, written consent was obtained. During the FGDs, participants were invited to use their real names or a pseudonym, and all participants were encouraged to keep the discussions confidential. Participants who attended the FGDs were provided with an information sheet giving details of organizations and instances with support services, should the discussions negatively affect participants’ wellbeing.

During transcription, all participant names and identifying information were removed. Transcriptions, recordings and informed consent documentation were stored on encrypted drives in a locked safe to which only the research team had access.

## Results

### Aware but confused: conflicted between individual and collective responsibility

The data analysis resulted in the development of one core category “Aware but confused: Conflicted between individual and collective responsibility”, which encapsulates the multifaceted tension in interpreting and interacting with the issue of sexual harassment in the context of Lund University. The analysis highlighted how confusion surrounding the continuum of individual-collective responsibility permeates multiple aspects of the process, from understanding and defining sexual harassment through setting the event in a broader context, contemplating one’s own place in this context and considering if and how to respond to the situation. The core category also has implications for questions of accountability and redress.

**Figure 2.**
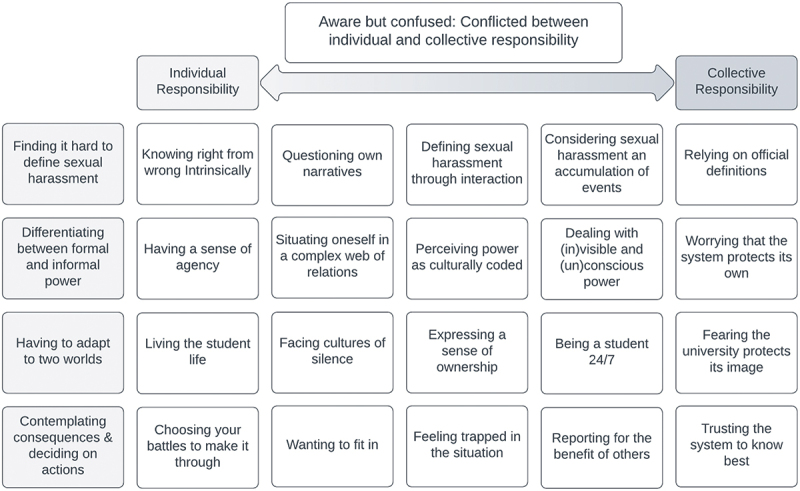
A theoretical model showing the links between the core category, main categories and sub-categories.

Figure 2 provides a visual overview of the theoretical model developed in the analysis, showing the core category “Aware but confused: Conflicted between individual and collective responsibility”, supported by four main categories, “Finding it hard to define sexual harassment”, “Differentiating between formal and informal power”, “Having to adapt to two worlds”, and “Contemplating consequences and deciding on actions”. The sub-categories further clarify and specify the categories and are presented with properties and dimensions along the continuum from individual to collective responsibility.

In the next section, the findings are presented in more depth, with the main categories shown in bold and sub-categories in *italics*. Quotes have been selected to illustrate how the interpretations are grounded in the discussions.

#### Finding it hard to define sexual harassment

This first category comprises aspects of what sexual harassment meant for students in the university setting, as well as discussions of who should be allowed to define such actions and their implications. The narratives in this category showed a broad range of opinions and views and a general conceptual disconnect where the same participant could hold multiple, sometimes conflicting, viewpoints.

FGD participants described how individuals *know right from wrong intrinsically*, and that sexual harassment was something that only the exposed person could define. Discussions in this matter were very gendered, with participants drawing dividing lines between the experiences of females and males and claiming that the latter were often not able to appreciate the severity of many events as they did not have previous experiences of harassment or sexual harassment to compare with.

My friends and I have grown up knowing clearly what is right and what is wrong. Ever since school we have known we have to think about these things every day … so I think we are much more aware than the boys are. (FGD6, Female)

At the same time, participants felt an urgent need to clarify definitions of sexual harassment and expressed confusion about where the dividing line was between sexual harassment and other forms of harassment. This knowledge about what was right and what was wrong was challenged by doubts about personal experiences and the severity of what they had been a part of, leading participants to *question (their) own narratives*. These narratives were often framed in uncertainty about the need to prove what had happened to them, and what sort of evidence would be sufficient. Challenges in distinguishing between sexual harassment and other forms of discrimination were illustrated through numerous situational examples where boundaries were discussed, especially in the context of international students from different cultures within the university. Participant stories showed a broad spectrum of opinions about the importance of defining events as sexual harassment, with opposing arguments about the irrelevancy of the discussion and the importance of challenging all offensive behaviour in a contextual manner.

For me it’s very tricky, for instance to draw the line between the sexual part of it (and harassment). I mean I’m not pretending that harassment is more positive than sexual harassment, but I think certain types of harassment that sometimes get categorised as sexual harassment can be just more broadly categorised for instance as gender-based harassment. (FGD1, Male)

Through the intertwined discussions of the participants a narrative emerged of sexual harassment being *defined through interaction*, with both a perpetrator and victim involved. In some situations, this revolved around the question of intentionality and whether differences in experience between victim and perpetrator were justified and important for the question of SH. Acknowledging differences in interpretation of situations was also reflected in narratives about how the same situation can be understood differently in different contexts. Also in this sub-category were those narratives where sexual harassment was defined as a repeated action requiring a clear “no” from one partner to fall under the definition. Understanding sexual harassment as an interaction broadens responsibility and raises a need for empathy and understanding.

Somehow, I think it is repeated. Like it’s not just about a hand in the wrong place in a club … I think that is crude behaviour, a wrong behaviour, but maybe not sexual harassment. But when it is repeated and happens again … then I think it is something. (FGD2, Female)

That it is repeated is something I agree with. I have seen things happen, not physical things but like repeated propositions party after party (…) where the person in question just won’t except a no. (FGD2, Male)

*Considering sexual harassment as an accumulation of events* was an argument that viewed sexual harassment as a cumulative phenomenon where each new individual event was interpreted in the context of every other event that had happened before. This narrative combined elements of the individual definition of sexual harassment and the shaping of sexual harassment through interaction with the claim that the repetition of an event need not be from the same person. Thus, a seemingly single event can be interpreted as one in a series of repeated events.

And it can have happened multiple times but from different people. So like there are many different people who do the same thing to one person. So even though each of them has only done it one time (FDG1, Male)

So even though each one of them has only done it one time it feels like you have been exposed more times. (FGD1, Female)

The narratives that ascribed most collective responsibility to the university setting were those that were *relying on official definitions* and emphasized the need for definitions that clearly provided guidance on these questions. Participants discussed not knowing what the university rules were, or how to interpret them. For these students there was an additional concern that the existing definitions in theory seemed to disappear in concrete cases that they had heard about.

In a university setting people are very good at analysing things in theory … and people can be very, very like aware of issues … but like actually implementing like strategies for dealing with sexual harassment that’s like another area, that’s like who, who is responsible for this, we don’t know. (FGD5, Female)

#### Differentiating between formal and informal power

This category reflects discussions around the spectrum of power and its role in forming, supporting, justifying and hiding sexual harassment in the university setting. It also mirrors considerations around the visibility and formality of power, connections between power and cultures/norms, as well as fears that those with positions of power could be tempted to use it to protect each other.

In general, students expressed *having a sense of agency* in their own activities and that student life was shaped by students and that they could therefore bring about change. At the same time, they were aware of their own position as *situated within a complex web of relations.* This complex web encompassed formal hierarchies as determined by the university systems, such as being employed vs being a student, or those with positions within student associations and “nations” but also informal power connected to being an older student, having access to information or resources through one’s social networks and even through being popular within the student body.

Some students have positions where they supervise or help younger students in the course. And they have a certain type of power there, it can be through access to knowledge, information, books, experiences about course. (FGD1, Female)Yes, and a big contact network. (FGD1, Female)Exactly, and they can be like ‘This exam is difficult, study harder on these parts’, it automatically becomes a power dynamic. (FGD1, Female)

Power was seen as “absolutely important for sexual harassment” (FGD7, Male) with misuse of power or consequences of power imbalance discussed as one of the primary causes of sexual harassment. Some of the positions of power were explained as being connected to different cultures and traditions at the nations or within the university system, or within broader society such as connections to gender inequalities and patriarchal structures. Within this argument of *perceiving power as culturally coded,* participants viewed the university setting as a space defined through power relations.

I think there are also many situations in the student social life where, I don’t want to say they incentivise, but they increase a sexualised environment or other sexual context through something as simple as traditions and norms that make a culture. (FGD4, Male)

Power in all its forms was discussed as having both *(in)visible and (un)conscious forms*. That power can be invisible to those who hold it, while being tangible and obvious to those who experience it was discussed in the context of not seeing sexual harassment or how it could be linked to expressions of power.

Because it is them, the people with positions of power who have influence over what you can say, the social norms, and they also have the opportunities to commit sexual harassment. (FGD1, Male)

Likewise, power as being unconscious was linked to the idea of intentionality, where even though people may accept that they have positions of power, they are not always aware of when this power is being enacted or what consequences it has.

I think it’s not just because you really have power. So you don’t intentionally use your power, or your position to do anything. But just being in that position. It doesn’t matter what you are capable of, but people are aware of those capabilities and that can lead to consequences. Not necessarily like the professor wants to do anything intentionally or wants to utilise that power to do anything. (FGD2, Female)

Those with power were more easily able to commit sexual harassment, to hide sexual harassment when it had occurred and to avoid the consequences of sexual harassment. This was especially felt in cases where those with a duty to examine and respond to sexual harassment cases were part of the same power structures as the perpetrators. These discussions were captured in the sub-category *worrying that the system protects its own*. Both among staff and students in positions of authority there was a fear that reports of sexual harassment would be met by dismissive statements such as “Oh I know that person” or “He’s not like that” (FGD1, Female) and that these reports would not lead to any concrete actions due to protection by friends and colleagues.

I feel there are far too many staff that protect one another and students too. There are too many unprofessional relations. It’s like you need to be a different person when you are working than when you are outside work. (FGD6, Female)

Exactly, when it comes to students and staff the other staff might not want to see that their friends have done something, so they protect each other and don’t believe the reports. (FGD6, Female)

#### Having to adapt to two worlds

This category illustrates a divide between students’ interpretation of sexual harassment in their study environment and in their student social life. However, it also clarifies an exchange between these two worlds and the ramifications of responsibility that follow.

In certain ways, students saw themselves as *living the student life*, which included all activities outside of formal teaching situations. This distinction between study environment and social environment translated into students seeing the university as having a specific role in dealing with sexual harassment in the university setting, but the social environment requiring action from students for students. The role of student organizations including the unions, student health services and the “nations” was seen as important in framing understandings and actions related to sexual harassment. There was a level of trust in student organizations beyond that reported for university structures. There was also a sense of the student scene being problematic, with stories of harassment and sexual harassment across a variety of situations while at the same time, participants reported feeling that the student life was safer than general society.

Yeah, because I personally I feel like there is a lot less sexual harassment within the student life than I’ve experienced in general society overall. I don’t know if you agree, but that’s my personal experience that I’ve never been sexually harassed in a nation, but I was sexually harassed like in a normal club like multiple times, so I feel like they are better in a sense. But I don’t know, they might be different from person to person as well. (FGD3, Female)

When contemplating the possibility for students to discuss sexual harassment with fellow students and university staff, participants described positive experiences about the opening up of the discourse and availability of language for talking about sexual harassment. Participants raised #MeToo as a contributing factor in this positive development. Despite this, some situations were still described as being characterized as a *culture of silence* where discussing sexual harassment openly was not even considered. This was present in student life, as well as in certain teaching moments, and was identified as one of the major obstacles to creating a protective environment at the university. The culture of silence discussion was also highly gendered, with female participants mentioning that they were able to discuss more openly with other females than with their male friends, especially where more than one male friend was present.

I think that in the case of friends it depends on the gender because I have a group of female friends and a group of male friends and when I talk about sexual harassment with my male friends many of them don’t know what to say, how to react, they are like okay, they don’t know what to do. (FGD3, Female)

I feel like it’s quite different in my group of friends(…) we talk about this stuff all the time and even if there is one guy friend amongst the group of female friends, we would talk about it and have discussions and I have had like guy friends share experience with us and stuff but as soon as there is like a couple of guys, the whole room changes. (FGD3, Female)

Participants in this study reported *expressing a sense of ownership* over the university space. This *ownership* was important for how they interpreted the limits of what they would accept and what consequences they would be willing to face. Participants who felt a strong sense of ownership of the university space reported being surprised when sexual harassment happened as they expected others to share their sense of responsibility. At the same time they mentioned that a sense of ownership also came with a sense of accountability and that this could encourage them to report what had happened to them to protect their spaces.

If someone in town shouts ’nice butt’, what can you do? You can’t go to the police with that. But here, this is more of our own space, because here is where I have my education. I have the right to be here. Town on the other hand is more like everybody’s space. (FGD 6, Female)

Although participants could sometimes differentiate between student life and social life, the category *being a student 24/7* captures those times when they felt the boundary between the two was fluid or even non-existent. Seeing the spill-over of student life into study situations, or the spill-over of group work problems into the students’ social life gave voice to some of the complex power dynamics in student life, as well as the potential complications and complexities in reporting sexual harassment. The fact that students often live together outside of student activities was also highlighted as one of the factors contributing to this lack of distinction. Framing students as always students shifts the responsibility of the university to encompassing all situations within and without university premises, a situation welcomed by participants but also problematized for its lack of practicality. This issue was in some ways made worse by the existence of online cultures where rules for behaviour were not as clearly established and jurisdiction not obvious. In these online environments, being a student 24/7 meant that you could not escape from any potential perpetrators.

I mean it’s all really a big bowl of soup but like a culture- the university and the dormitory and the students, so if anything, it would be foolish not to at least consider it”. (being one and the same) (FGD7, Female)

Like people are sitting at home studying and they are social online so I think a lot of harassment can come from there also through the phone, talking on the phone, sending pictures and stuff. (FGD7, Female)

While the participants discussed the need to report sexual harassment, there was a clearly articulated distrust in the university setting, captured in the idea of the *fearing the university protects its image*. Lund University was portrayed in the discussions as a prestigious and elite university that attracts highly competent students and staff. While some felt that this should make the university more open to discussing sexual harassment due to the collected expertise and clear responsibility for students’ wellbeing, others saw embedded traditions and cultures of silence upheld by university structures that diminished opportunities for accountability. The university staff were seen as protecting one another and the university’s reputation, and the students reported choosing to work with student organizations or directly with the police to avoid the university system they saw as defensive and sometimes dismissive.

I think the (university’s) reputation has a big stake in a lot of decisions or not taking decisions about putting measures against sexual harassment in place, but it might be that the person who is in power, it’s too much of a burden to take measures even though in the long term it’s actually for the better of everyone because I don’t think people in power would be comfortable knowing that there are problems. (FGD5, Female)

#### Contemplating consequences and deciding on actions

The last category clarifies how decisions on what actions to take is intrinsically intertwined with considerations of what the consequences could be for the individual, their social scene, academic progression and career. It also indicates how students view their responsibility for others at the university and future generations.

The pragmatic and individualistic approach for dealing with experiences of sexual harassment is encapsulated in the sub-category *choosing your battles to make it through*. Here participants talked about doing whatever gave them the easiest life and deciding sometimes to just ignore what has happened because it seems the easiest way to deal with the issue at the time. Participants spoke of questioning the seriousness of the situation in relation to potential consequences, and how the perpetrator’s position could also influence the decision to take action or not.

I have had some amazing evenings out in different clubs and at balls and lots of fun activities, but it can still happen that someone touches you inappropriately or says something inappropriate. But you choose to ignore it because you’d rather have a nice evening and it’s a bit of a sad situation to be in so you don’t deal with it and try to fix it just because it is such a pain to deal with these things and it will ruin the atmosphere. (FGD4, Female)

Deciding to live with it or ignoring what has happened was intertwined with an acute fear of social isolation and participants’ *wanting to fit in* with the university setting, especially with the social scene at the university. This discussion highlighted a number of groups who were seen as more vulnerable to the pressure to conform based on their youth, status as international students or “newness” in a situation. These three factors were discussed as making students experience greater peer pressure to accept what was happening in an attempt to fit in. In these cases, students often chose not to take any action and simply accept what has happened.

I have thought about this a lot, and I felt that I needed to fit in, and like figure out how I behave in this environment and what is ok and not ok, especially as a new person there. (FGD1, Female)

Personally I feel like this, it took time. I didn’t really understand what was happening at the start and it took time before I could understand what had happened and sort it into the category of sexual harassment. (FGD1, Male)

*Feeling trapped in the situation* was a shared perception in all group discussions. This included fear of continuing to see the perpetrator in academic and social environments and fear of the impact of any action on their grades, future career and social networks. Participants reported that being trapped was often connected to relationships of power and dependency and made it less likely that they would take further action if sexual harassment has occurred. A tangential narrative also emerged where some students felt compassion for the perpetrator and felt that reporting events could lead to serious consequences for the perpetrator that outweighed the event that precipitated them, thus contributing to their feeling of being trapped.

Hopefully when you report the university will actually do something about it, but what that could be I don’t know. I think people are uncomfortable about having to meet the person again. If it happens during a course, you have to finish the course to get your certificate and then you have to keep meeting the person. (FGD6, Female)

Similarly, students expressed hesitation at the thought of reporting as the consequences for themselves were considered to be serious. At the same time, stories emerged of those who had chosen to report on behalf of others. This view of *reporting for the benefit of others* was sometimes framed as protecting the next generation of students, ensuring that this did not happen to someone else. Thus, even when the consequences were deemed serious for the individual, taking a broader perspective was highlighted as a meaningful and praiseworthy cause. Reporting even minor events for the sake of building up an evidence base was also discussed at length.

I am torn, it depends on like what reporting will lead to, what is best for me? And then like am I reporting so that others don’t have to be exposed to this or am I just doing it for myself. I think that often you feel that this shouldn’t happen again and that is what drives a person to report. (FGD1, Female)

Choosing to report sexual harassment was seen as a complex balancing act between interpreting the situation, trusting in one’s own narrative, considering the consequences and finally deciding where to take action. Despite this convoluted path, participants in this study placed great value on creating a university culture where they could *trust the system to know best.* Students felt left out by the university system in its current form, questioning how the investigation actually looks, and whether there are significant results to reporting. This lack of trust and clarity in the current situation was juxtaposed against a hope that the system could become the system that they want, and that this would be the best solution in the long term to address the reporting and management of cases.

The main thing is, as I said earlier, it’s about a system. I mean of course people play a role, but the university has to show me that there is a system that is solid, it’s transparent, it’s clear and it’s visible (…) If there was a concrete system then if something went wrong, I could do something about it. (FGD7, Male)

Maybe make an effect to create like a culture where it feels like it- like the university cares about these things. (FGD7, Female)

Yeah, that was also what I was thinking like. The crucial point I think would be to repower (victims), a system that is caring and gives you back power and control. (FGD7, Male)

## Discussion

The findings of this study highlight the contradictions faced by university students in experiencing and responding to sexual harassment that are captured by the core category Aware but confused: conflicted between individual and collective responsibility. This contradiction permeated every aspect of understanding sexual harassment at the university. The process starts with students *finding it hard to define sexual harassment*. Despite being aware of sexual harassment at the university, students were torn between an individual’s experience, an interaction event and official definitions. This in turn is influenced by discussions of *formal and informal power* relations and the feeling of having to *adapt to two worlds* as students in their studies and in the student social life. The conflict between individual and collective responsibility was also clear in discussions of consequences of sexual harassment, consequences of reporting sexual harassment and how one should think about *consequences and deciding on actions.*

Despite an awareness of the existence of sexual harassment and of some elements of the university’s response to it, students conceptualize, rationalize and motivate actions based on interpretations of the phenomena of sexual harassment on multiple levels. The analytical model generated in this study can be viewed as an empirical exemplification of the four levels of analysis model developed by Doise (Doise, 1986) in the specific context of a university setting. To obtain a broader understanding of the phenomena and to examine the usefulness of the developed analytical model (Charmaz, 2014), the discussion will therefore be structured around the four levels of analysis, with each level introducing a conceptualization of sexual harassment grounded in the results of this study, before contextualizing to other research and perspectives.

## Intraindividual level

The intraindividual level of analysis captures the way individuals process and organize their perceptions of the social environment (Doise, 1986). In this study, intraindividual conceptualizations of sexual harassment regarded it as an individual experience that is shaped by one’s own perceptions and beliefs. Participants discussed how they could know right from wrong intrinsically, and that the definition of an event as sexual harassment was up to the person who had been exposed, independent of other factors. This conceptualization shares similarities with research into the concept of self-labelling, and the role of cognitive dissonance in labelling behaviour as sexual harassment (Magley & Shupe, 2005). In this study, these discussions revealed differences in how males and females understood situations as sexual harassment. Research into sexual harassment that is guided by an intraindividual level of conceptualization tends to focus more on the perpetrator and emphasizes the internal dialogues and mental concepts of sex and power held by individuals that have been shown to increase the likelihood to sexually harass (Pryor & Stoller, 1994). Although the results of this study did identify individual and internal elements of sexual harassment, such as intrinsic knowledge of right and wrong, the group discussions did not focus on perpetrator characteristics, with discussions instead highlighting the perspectives of the person exposed to harassment. This is in contrast to much of the existing literature and its focus on perpetrator traits.

Other qualitative research conducted in Sweden has examined how individual conceptualizations of sexual harassment can make formulating a response complex, in that a lack of clarity can mean that “everything and nothing” can be considered sexual harassment (Carstensen, 2004). This is supported in the current findings where students expressed uncertainty with regard to identifying and acting on sexual harassment. The individualization of sexual harassment can also locate the responsibility of dealing with sexual harassment on an individual level and neglect the responsibility of structures and groups, a finding of our study that supports and expands upon previous findings from a study in the UK (McCarry & Jones, 2022). Moving towards a more collective framing of sexual harassment has also been shown to broaden engagement in sexual harassment prevention programmes by appealing to students beyond those who have personal experiences on an individual level (Graham et al., 2021).

### Interindividual level

The interindividual level of analysis is characterized by the dynamics of relations among individuals and between individuals and groups (Doise, 2012). This situational approach studies cognitive processes as embedded in or generated by different conditions. In this analysis, the need to validate one’s experiences through discussing with friends, and thus make explicit what one has experienced, is a conceptualization of sexual harassment on an interindividual level. This conceptualization shares similarities with attribution theory, more specifically the construct of consensus where the experience of others of a situation is used to calibrate an individual’s own perception of that situation (Klein et al., 2011).

The current findings also highlight the gap between a theoretical understanding of sexual harassment and an application of this understanding in the interpretation of personal experiences as sexual harassment, findings echoed in recent qualitative research conducted in Sweden (Hagerlid et al., 2023).

Sexual harassment as an interaction was also reflected in how some participants reasoned around sexual harassment as requiring an event to be repeated, and an exchange to occur between perpetrator and victim to establish limits and be clear about when a boundary has been crossed. This reasoning was challenged, however, by other participants during the course of the discussions and is not supported by previous studies outside of the university setting that show that in hypothetical situations, each sexual harassment event is interpreted independently from those that have previously occurred (Marks & An, 2019).

The concept of an event requiring a shared understanding to be considered sexual harassment by both parties could also explain how many instances of sexual harassment can be conducted “unconsciously” by the perpetrator where they do not share the same experiential framework as the victim (Fitzgerald et al., 1988).

In facing new situations in “nations” social clubs or other group constellations, students were also forced to re-calibrate their understandings and expectations to account for group norms and dynamics. The perceptions that peer attitudes and behaviours help create an environment where sexual harassment can occur confirm research conducted among college students in the USA who identified this issue (Bonar et al., 2022). In the same study, addressing peer norms and group interactions was shown as a way to break down “cultures of silence” and increase reporting (Bonar et al., 2022). The current analysis also reflected elements of this through students’ suggestions to break down microcultures and protect new students as a way to challenge entrenched sexual harassment. In a systematic review of literature published in the US and Canada, Mujal and colleagues analyse interventions that have used bystander approaches to combat sexual harassment (Mujal et al., 2021). Similar to the suggested results of this study, bystander approaches were appreciated by the participants and were found to be an effective way to improve attitudes and behaviours regardless of setting.

### Positional level

Analyses on the positional level concern the positions occupied by individuals and social categories in a given context. On this level, conceptualizations consider the influence of social status, power and the asymmetrical value placed on specific groups in society that reinforce expectations and roles (Doise, 2012).

In this study, both conceptualizing sexual harassment through interaction and making the decision whether to report perceived sexual harassment or not were seen as being contingent on the positions that the perpetrator and victim hold as well as the position of the person to whom you must report. Complex interplays of power dynamics and social status afforded by them in the university and social contexts had far reaching effects for how students in this study perceived and discussed acting on sexual harassment. These positions could be formal, as in the case of those with positions of authority granted by office, or informal in the sense of students with access to social networks, knowledge or information.

These findings are in line with the large body of research conducted in workplaces that build on organizational theories and examine the relationship between power and status within organizations and sexual harassment. The importance of lifting the focus from being a student problem to being a problem that can be understood in a “conducive context” through structural mechanisms is similar to the findings of a study conducted among students and faculty in the UK (McCarry & Jones, 2022). In similarity with these findings, in workplaces in the US, direct power (in form of supervisory roles) was shown to both enable sexual harassment to occur, and to increase the severity of the sexual harassment act (Cleveland & Kerst, 1993). Likewise, in this study, sexual harassment that occurred down a power gradient was deemed as more problematic by the study participants, especially in the case of formal positions of power such as teacher-student relationships. This was partly due to the easier task of identifying the behaviour as problematic, and partly due to the perceived consequences of such harassment. These findings could also be compared to the concept of dignity, and especially to social dignity as described by Jacobson (Jacobson, 2009). In her grounded theory study conducted among individuals marginalized by health or social status and those supporting these individuals, she presents a taxonomy of dignity in which social dignity is conceptualized and discussed as a social and interactive process. The clear connections to power, organizational culture and consequences of violation of dignity bear strong resemblance to the positional ideas of this study (Jacobson, 2009).

Some researchers of organizational theory suggest that the very act of sexual harassment can be an attempt to equalize power in the organization (so called contrapower harassment) (Cleveland & Kerst, 1993; McKinney, 1992). This aspect was, however, not reflected in the narratives of the participants in this study.

Power in this study was also discussed as having invisible and unconscious forms. The idea of unconscious sexual harassment has been examined from a social cognitive approach on intrapersonal levels where the interconnectedness of the power-sex association in individuals renders them unaware (Pryor & Stoller, 1994). This approach has been criticized as having a “poor explanatory depth” (Pina et al., 2009). Other research has examined sexual harassment in the workplace where supervisors do not intend to use their position/power to initiate a response, but the subordinates’ perception of the inherent power difference can lead to this interpretation (Fitzgerald et al., 1988).

A separate argument for the unconsciousness of sexual harassment among men problematized the idea of male ignorance and instead considered the idea of a voluntary “not-knowing” as a way to “cultivate, curate, and coordinate” systemic privilege (O’Neill, 2022). These theories can also be linked to concepts of epistemic injustice, understood as prejudice that diminishes the credibility of a speaker and can lead to the systematic disadvantaging of certain groups (Coady, 2010). In the context of sexual harassment this phenomenon has been used to theorize around the invisibility of sexual harassment (especially for men) and the role #metoo has had in challenging this phenomenon (Jackson, 2018). In this study participants attributed positive developments to the #metoo movement but highlighted the continued challenges in dealing with dominant masculinities as discussed on the ideological level.

Research into the reporting of sexual harassment in the workplace has highlighted the key role that organizational climate plays (Bergman et al., 2002). This has been understood as the need for psychological safety, which represents a work context within which one feels safe to express oneself without fear of negative consequences (Singletary Walker et al., 2019). Discussions of psychological safety are closely tied to power and dynamics and consequences of reporting. Other research conducted at Lund University has highlighted the important role that supervisor and lecturer support can play in modifying the association between sexual harassment and stressful study environments and supports the important role that those in power have for creating a supportive environment (Palmieri et al., 2023). The results of this current study expand that understanding by raising fears of collegial protectionism that would need to be overcome and a desire for a system that embodies the empowerment of victims throughout the reporting process.

### Ideological level

The ideological level of analysis largely focuses on belief systems, social representation and norms of a given society (Doise, 2012). In this study, permeating the previous three levels of analysis and found throughout the analytical model are questions about the role of cultural norms and beliefs that reinforce values that can lead to sexual harassment.

Female participants expressed still experiencing hesitation from their male colleagues about discussing sexual harassment and the persistence of cultures of silence. This supports the findings of McCarry and Jones (McCarry & Jones, 2022) whose research into sexual harassment and gender inequality in UK universities highlighted the “invidious circle” feedback loop between these two concepts.

Another conceptualization of sexual harassment on the ideological level was an articulation of traditions and norms embedded in the student and university cultures that justified and perpetuated situations where sexual harassment can occur. Developed as an extension of research examining rape myths, legitimizing myths about sexual harassment have been shown to provide justification for social practices that distribute social value within a system (Sidanius & Pratto, 1999). These systems need to be supported by different positions within the group hierarchies including those newest members (Augoustinos et al., 2014). These notions were supported in this study where new and younger members in the various social contexts felt pressured to adapt to the existing structures and, in their turn, recreate them.

### Interconnection between levels

The articulation of the four levels of understanding of sexual harassment provided here can be instrumental in appreciating both the complexity of the topic and the sense of confusion reflected in the students’ narratives. The results of this study show that far from being an individual experience, sexual harassment in the university setting is conceptualized in the context of perceived organizational norms and structures. The analytic model illustrates the interconnectedness between the different levels of understanding through the interplay between the individual and the collective realms of responsibility. In seeking to address this ambiguity, the university should provide clarity on the system level with regards to definitions, structures and consequences of reporting sexual harassment. This could allow individuals to navigate a path through the discussion of individual and collective responsibilities and allow the university to acknowledge the role it has in challenging harmful cultures and changing damaging structures.

### Methodological considerations

The ability to judge trustworthiness in qualitative research relies in large on transparency and reflection on how data were collected, analysed and presented to address the research question (Dahlgren et al., 2007). Several steps were taken to improve the trustworthiness of this study.

Firstly, all authors of the article have been employees of the university and two of the authors (JP and PS) have been students at the university. This gave the authors familiarity with the university setting and an ability to create rapport during the group discussions. At the same time it required reflexive approaches to ensure that all authors were aware of how their own backgrounds might shape data analysis (Charmaz, 2014), with an aim to reflectively interpret, not eliminate bias caused by researchers’ views (Corbin & Strauss, 2015).

Secondly, the group discussions were advertised widely across the university and all students invited to take part. Although the sample is small, participating students came from a variety of faculties and campuses and represented a range of perspectives. The selection of FGDs for data collection was motivated partly by the aim of exploring organizational structures and norms, and partly by their appropriateness for discussing sensitive topics through encouraging group interaction and allowing multiple points of view to be expressed and reflected upon (Wear & Aultman, 2005).

Finally, applying Charmaz’s trustworthiness criteria (Charmaz, 2014), the constant comparison method was adopted to ensure sufficient and relevant data and increase credibility, and quotes used to exemplify how the analysis was grounded in the data. Memo-writing and peer-debriefing between the researchers were an integral part of the process and reflect a reflexivity in the analysis and data collection. The use of theoretical sampling (Charmaz, 2014) supported the resonance of the results.

The results of the study extend and support many of the findings in existing research conducted in other settings, and the analytical model serves as a framework for discussing different conceptualizations of sexual harassment and their impacts on what actions are perceived as possible.

## Conclusion

Students in the university setting of this study were aware of the occurrence of sexual harassment and the existence of university policies to prevent and manage cases. At the same time, they were confused about how to conceptualize sexual harassment in this setting and where responsibility for prevention and management lies on a continuum from individual to collective. This confusion could have serious consequences for willingness to report cases of sexual harassment. This study further suggests that building trust in the university system requires clarity in a number of key areas. These include establishing common understandings of sexual harassment, clear and accountable pathways for reporting sexual harassment and transparency of outcome when reports are made.

## Data Availability

The participants of this study did not give written consent for their data to be shared publicly. Due to the sensitive nature of the research therefore, supporting data is not available.
